# CRISPR Genome Editing in Personalized Therapy for Oral and Maxillofacial Diseases: A Scoping Review

**DOI:** 10.3390/biomedicines13112745

**Published:** 2025-11-10

**Authors:** Arkadiusz Dziedzic, Robert Kubina, Magdalena Skonieczna, Marcel Madej, Jakub Fiegler-Rudol, Mushriq Abid, Doaa Nadhim, Marta Tanasiewicz

**Affiliations:** 1 Department of Conservative Dentistry with Endodontics, Medical University of Silesia, 40-055 Katowice, Poland; martatanasiewicz@sum.edu.pl; 2 Department of Pathology, Medical University of Silesia, 41-200 Sosnowiec, Poland; rkubina@sum.edu.pl; 3 Department of Systems Biology and Engineering, The Silesian University of Technology, Akademicka Street 16, 44-100 Gliwice, Poland; magdalena.skonieczna@polsl.pl; 4 Biotechnology Centre, Silesian University of Technology, Krzywoustego Street 8, 44-100 Gliwice, Poland; 5 SilesiaLab, Research & Development Centre, Medical University of Silesia, 40-055 Katowice, Poland; marcel.madej@sum.edu.pl; 6 Department of Periodontal Diseases and Oral Mucosa Diseases, Faculty of Medical Sciences in Zabrze, Medical University of Silesia, 40-055 Katowice, Poland; jakub.fieglerrudol@gmail.com; 7 Department of Orthodontics, College of Dentistry, University of Baghdad, Baghdad 01110, Iraq; 8 Department of Periodontology, Ministry of Health, Baghdad 01110, Iraq

**Keywords:** gene editing, CRISPR, Caspase-9, oral medicine, oral pathologies, craniofacial defects, genetics, oral cancer, targeted therapy

## Abstract

**Background:** CRISPR/Cas genome editing is emerging as a powerful tool in oral and maxillofacial medicine, with potential applications in personalized therapies for conditions that currently lack durable treatments. **Objectives**: This scoping review aimed to map existing evidence on CRISPR-based applications in oral and maxillofacial fields, rather than to assess treatment effectiveness. **Methods**: A systematic search of PubMed, Scopus, Web of Science, and ClinicalTrials.gov (2012–2024) identified studies and registered trials involving CRISPR with oral health relevance. Eligible articles included peer-reviewed experimental reports and clinical trials. **Results**: From 1437 records, 121 studies met inclusion criteria: 106 preclinical reports and 15 clinical or translational studies. Investigated domains included oral cancer therapy, hereditary craniofacial syndromes, regenerative strategies, infectious disease models, and pathogen detection. Early clinical efforts focus mainly on CRISPR-edited T-cell immunotherapies in oncology. Major barriers include off-target effects, delivery challenges, regulatory complexity, and ethical concerns. **Conclusions**: CRISPR-based bioengineering shows strong promise for precision care in oral and maxillofacial medicine. However, current evidence remains largely preclinical and heterogeneous. No clinical recommendations can yet be made, and translation will depend on rigorous late-phase trials, ethical oversight, and health-economic evaluation.

## 1. Introduction

In recent years, advancements in genetic engineering have significantly impacted the diagnosis and treatment of a wide range of diseases, including those affecting the oral and maxillofacial region [1,2]. Oral and maxillofacial diseases represent a major global health burden, with oral conditions such as untreated dental caries, severe periodontitis, and edentulism affecting over 3.5 billion people worldwide, according to the Global Burden of Disease Study. These conditions not only compromise oral function and quality of life but also contribute to systemic health risks, underscoring the need for innovative and personalized treatment strategies [1,2,3,4,5,6,7]. Among these innovations, CRISPR (Clustered Regularly Interspaced Short Palindromic Repeats) technology has emerged as a precise and cost-effective gene editing method with the potential to revolutionize modern medicine, including oral healthcare [3,4,5,6,7]. CRISPR/Cas systems allow for the targeted modification of DNA, enabling the correction of pathogenic mutations, suppression of disease-causing genes, and enhancement of diagnostic tools. This has sparked growing interest among researchers and clinicians in the fields of oral medicine, maxillofacial surgery, and oral pathology [4,5]. Applications in these areas include treatment strategies for hereditary craniofacial syndromes, oral cancers, autoimmune diseases, and microbial infections, many of which have historically lacked effective long-term therapies [6,7,8,9]. A key strength of CRISPR lies in its ability to induce targeted DNA cleavage with high efficiency and accuracy through the Cas9 endonuclease and guide RNA system. This process, based on homology-directed repair (HDR) or non-homologous end joining (NHEJ), can lead to either correction or silencing of specific genes [10,11,12]. However, concerns remain regarding off-target mutations, unintended insertions or deletions (INDELs), and unpredictable DNA repair outcomes [13,14]. These technical limitations, along with ethical and regulatory issues, represent important barriers to clinical translation [15,16]. Despite these challenges, there is a growing need for novel, personalized therapeutic approaches in oral and maxillofacial disciplines. The use of programmable molecular technologies such as CRISPR may offer viable alternatives to conventional treatments, particularly in cases where current interventions fail to provide durable results. This review explores the current and emerging applications of CRISPR genome editing in oral medicine, identifies limitations, and highlights future opportunities within this rapidly evolving field.

This review specifically focuses on mapping evidence relevant to oral medicine and maxillofacial surgery, where emerging genetic tools remain underexplored compared with systemic medicine. A scoping review was selected to capture the breadth of CRISPR applications in oral health, identify knowledge gaps, and contextualize translational barriers unique to dental and craniofacial fields. This review explores the potential applications of CRISPR-based genome editing and its anticipated impact on the future clinical management of various oro-facial diseases and pathologies. We highlight the remaining challenges that must be addressed before genome editing can become a viable therapeutic option, the opportunities for advancing CRISPR technology, and the ethical considerations that require careful attention.

While previous reviews have discussed gene editing technologies in general medicine or systemic oncology, few have specifically synthesized CRISPR-related evidence within the context of oral and maxillofacial diseases. This review adds novelty by systematically mapping the breadth of CRISPR applications across oncology, regenerative medicine, hereditary syndromes, infectious diseases, and diagnostics in dental and craniofacial care. Unlike earlier works, it distinguishes preclinical from translational evidence, highlights feasibility through technology readiness levels, and identifies oral health–specific barriers that are underrepresented in the broader CRISPR literature.

## 2. Methodology

This review followed the PRISMA-ScR (Preferred Reporting Items for Systematic Reviews and Meta-Analyses extension for Scoping Reviews) guidelines to ensure transparency and methodological rigor [17].

### 2.1. Literature Search Strategy

A comprehensive literature search was performed in four major biomedical databases: PubMed/MEDLINE, Scopus, Web of Science, and ClinicalTrials.gov, covering the period from January 2012 to November 2024. The search strategy used combinations of Medical Subject Headings (MeSH) and keywords including: “CRISPR,” “CRISPR-Cas9,” “gene editing,” “oral cancer,” “oral diseases,” “maxillofacial,” “craniofacial,” “periodontitis,” “dental”, “dentistry”, ”regenerative medicine,” and “clinical trials.” An example PubMed string was: (“CRISPR” OR “CRISPR Cas9” OR “Cas12” OR “Cas13” OR “base editing” OR “prime editing”) AND (“oral” OR “dent*” OR “periodont*” OR “maxillofacial” OR “craniofacial” OR “salivary”). Syntax was adapted for Scopus and Web of Science. Full database-specific search strings, including Boolean operators and MeSH terms, are provided in the Supplementary Material (Table S1) to ensure transparency and reproducibility of the search process.

Inclusion criteria were:

- Peer-reviewed articles and registered clinical trials involving CRISPR-based technologies;
- Studies relevant to oral medicine, maxillofacial pathology, or related systemic conditions with oral manifestations;
- English-language articles published from 2012 onward.
- Exclusion criteria were:
- Editorials, abstracts, conference posters, and opinion pieces;
- Non-human studies without translational application;
- Studies lacking mention of CRISPR/Cas or gene editing tools;
- Additionally, references of included articles and regulatory databases were manually screened for completeness.

### 2.2. Study Selection and Screening Process

**Figure 1:**
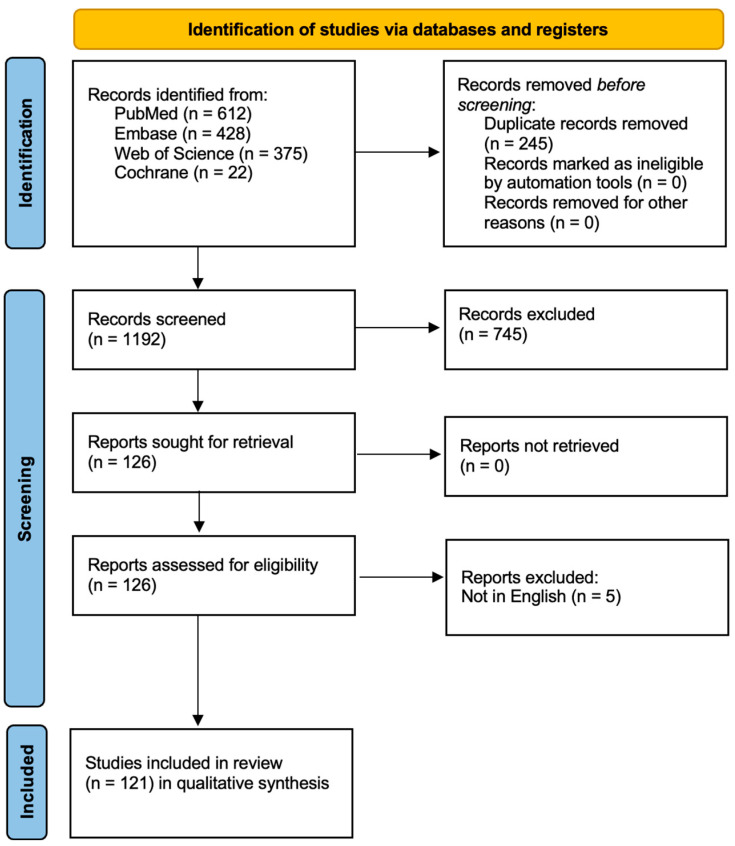
Study Flow Diagram.

After removing duplicates, titles and abstracts were screened for relevance. Full texts were assessed for eligibility based on inclusion criteria. The overall study selection process is presented in a flow diagram (Figure 1) for transparency.

### 2.3. Quality Assessment

For the registered clinical trials identified in this review, we performed a structured 9-item quality appraisal to support transparent interpretation of early-phase evidence. Specifically, we assessed whether each study was fully registered with a valid NCT identifier and protocol synopsis; whether the stated phase and objectives were appropriate to the investigational product; the clarity and completeness of the intervention description, including modality, target, and delivery; the accuracy and timeliness of the reported recruitment status; the definition of the target population and cancer type, with attention to inclusion and exclusion criteria; the presence of safety and ethical oversight, including IRB approval and independent data monitoring; the specification and relevance of primary and secondary outcomes, including on-target and off-target assays when applicable; the rigor of the study design, such as control strategy, dose escalation schema, and statistical plan; and transparency in adverse event reporting and reasons for withdrawal, termination, or completion. This focused appraisal does not estimate effect size but provides a consistent lens for comparing heterogeneous CRISPR-based trials and for contextualizing their translational relevance to oral and maxillofacial care. Based on the nine-item quality appraisal, these trials demonstrate moderate to high overall methodological quality, with individual trial scores estimated between 5/9 and 8/9. Feasibility and safety oversight are consistently strong, reflecting well-designed protocols for dose escalation, ethical monitoring, and clear intervention descriptions. However, their clinical applicability remains limited, as most studies are still Phase 1 or Phase 1/2, primarily focused on feasibility and short-term safety rather than demonstrating therapeutic efficacy or long-term outcomes. This indicates that while these CRISPR-based interventions are technically sound and promising, further late-phase trials are needed before they can be integrated into routine clinical practice. While no formal quantitative risk of bias tool was applied, we conducted a structured, qualitative appraisal of trial registration status, study design, safety oversight, outcome reporting, and methodological clarity. This approach supports trust in the synthesis by highlighting strengths and limitations of early-phase CRISPR studies without implying comparative effect size estimates.

### 2.4. Data Extraction and Synthesis

Key information extracted included:

○ Study type and design;
○ Disease focus;
○ CRISPR system used (e.g., Cas9, Cas12a);
○ Clinical setting or application (e.g., diagnostic, therapeutic, regenerative);
○ Summary of results or clinical endpoints (where applicable).

Given heterogeneity in design and outcomes, we did not perform a meta-analysis. We conducted a qualitative synthesis and evidence mapping. Findings were grouped by indication, tissue target, editing context, delivery vector class, and model maturity. We stratified results as preclinical, translational, or clinical and assigned an adapted technology readiness level for context.

This scoping review did not include a formal risk of bias assessment. The objective was to map the breadth of evidence that links CRISPR platforms to oral and maxillofacial applications rather than to estimate effect sizes.

A total of 1437 records were initially identified. After removing irrelevant entries, 1192 records were screened. Of these, 745 were excluded based on title and abstract, as they did not meet the inclusion criteria. Ultimately, 121 studies were included in the qualitative synthesis. Most included studies were preclinical, with limited direct clinical data for oral-specific CRISPR applications. Only a small proportion (n = 15) involved interventional clinical trials, primarily in oncology. No completed trial to date has directly evaluated CRISPR efficacy in common oral diseases such as caries or periodontitis. This evidentiary gap highlights the field’s early developmental stage. While CRISPR-Cas9 shows promise in regenerative strategies (e.g., salivary gland, enamel), most data come from animal models or iPSC-derived organoids. Translation into clinical protocols remains speculative.

## 3. Fundamentals of Gene Editing Biotechnology in Medicine and Biomedical Research

CRISPR/Cas9 technology has transformed gene editing practices due to its efficiency, ease of use, and adaptability. Compared to earlier technologies such as zinc finger nucleases (ZFNs) and transcription activator-like effector nucleases (TALENs), CRISPR requires fewer resources and allows for precise targeting of genomic sequences with a single guide RNA (sgRNA) [18,19,20,21]. In clinical and translational research, the most used system is CRISPR/Cas9, derived from Streptococcus pyogenes. This platform functions through a simple mechanism: a guide RNA directs the Cas9 endonuclease to a specific DNA sequence, where it introduces a double-strand break. The cell then repairs this break using either non-homologous end joining (NHEJ), which can introduce mutations, or homology-directed repair (HDR), which allows for precise correction using a template [22,23,24,25,26,27].

### Key CRISPR Types Relevant to Biomedical Use

**Figure 2:**
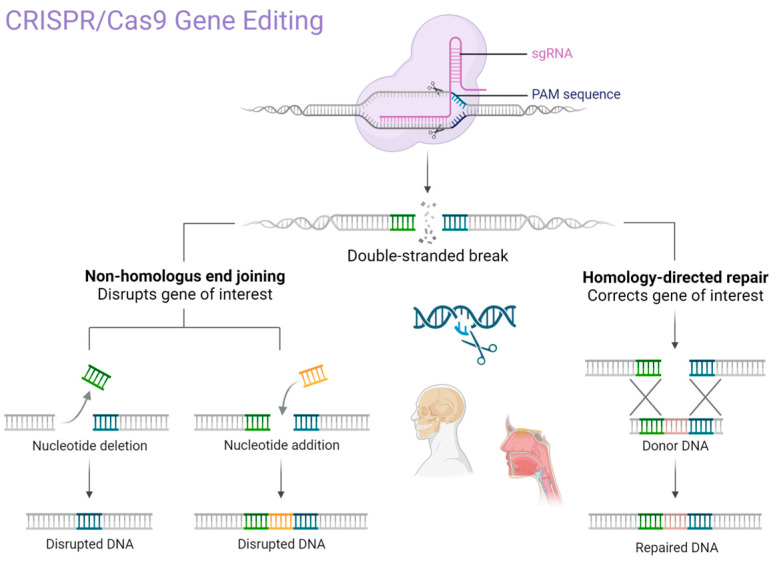
The primary mechanism of CRISPR/Cas9 gene editing technology. Created in BioRender. Dziedzic, A. (2025) https://BioRender.com/p62u5m9 (accessed on 1 February 2025).

The CRISPR system is classified into six main types (I–VI), each with distinct features. Cas9 (Type II) remains the most widely used in medical research due to its versatility and predictable activity. Other Cas proteins, such as Cas12 and Cas13 (Types V and VI), are increasingly being studied for DNA and RNA editing, respectively [28,29,30,31]. Given the technical complexity of enzyme-specific functions (e.g., HNH, RuvC domains), we summarize the CRISPR/Cas9 mechanism and domain activity in Figure 2 for clarity (Table 1).

## 4. Clinical Trials for CRISPR GEBT

In medicine, CRISPR/Cas9 GEBT provides therapeutic benefits primarily for diseases associated with gene mutations, e.g., hereditary diseases/disorders, cancer immunotherapy, other inherited immune system defects, and viral infections [32,33,34,35,36]. Nowadays, the pending clinical trials will deliver results with practical clinical implementation. These promising indications include various systemic diseases, primary hematological malignancies, in combination with another state-of-the-art technology chimeric antigen receptor T cells (CAR-T) (ClinicalTrials.gov, accessed 15 November 2024 [37]): allogenic anti-CD19 CAR-T cell therapy for relapsed/refractory hematological malignancies, T-cell lymphomas, B cell leukemia (chimeric antigen PACE CART19), multiple myeloma (NYCE T cells), metastatic gastrointestinal cancers, mesothelin positive solid tumors (*PD-1* and *TCR*-gene knocked out mesothelin-directed CAR-T cells), refractory viral keratitis, enterovirus infections (detection), pulmonary tuberculosis (rapid detection), severe sepsis (diagnostic tests), sickle cell disease, HIV-1 infection (modified CD34+), HPV-related cervical intraepithelial neoplasia, thalassemia, renal cell sarcoma, nasopharyngeal carcinoma (*PDL-1* knockout), including advanced stage IV, Rett syndrome (craniofacial malformations), esophageal cancer and associated malignancies, neurofibromatosis type 1 (oral manifestations frequently), Duchenne muscular dystrophy retinal diseases, amyloidosis, metabolic diseases, endocrine system diseases, autoimmune diseases. Table 2 summarizes the current and emerging clinical applications of CRISPR in oral and maxillofacial medicine, highlighting both the therapeutic and diagnostic domains. Table 3 illustrates that preclinical CRISPR applications in oral medicine show promising outcomes, from tumor suppression in murine oral cancer models to enamel and bone regeneration and modulation of periodontal pathogens, while Table 4 highlights the major ethical, technical, and economic barriers that currently limit translation into clinical practice [3,5,6,38,39,40,41,42,43,44,45,46,47,48,49,50,51,52,53,54,55,56,57,58,59,60,61,62,63,64].

Table 5 outlines future directions for CRISPR in oral and maxillofacial medicine, ranking applications by feasibility, with near-term promise in pathogen diagnostics, moderate potential in ex vivo regenerative strategies, and long-term but speculative prospects for in vivo gene therapy in hereditary craniofacial disorders. Feasibility was judged qualitatively, by reviewer consensus, consistent with a scoping review. For each application, we weighed: biological plausibility, delivery practicality in oral and maxillofacial settings, safety signals (off-targets, immunogenicity), evidence maturity across models, and implementability (workflow, regulation, resources). We assigned three levels: High-convergent preclinical or early clinical data with workable delivery and acceptable early safety; Moderate–good rationale with gaps in delivery, safety, or replication; Low–concept-stage with major unresolved barriers. We also mapped each area to an adapted TRL scale: TRL 1–3: early research; TRL 4–6: validation or early human study; and TRL 7–9: clinical demonstration to adoption. No formal risk of bias assessment was performed.

## 5. The Potential Application of Gene Editing Biotechnology in Personalized Oral Medicine

**Figure 3:**
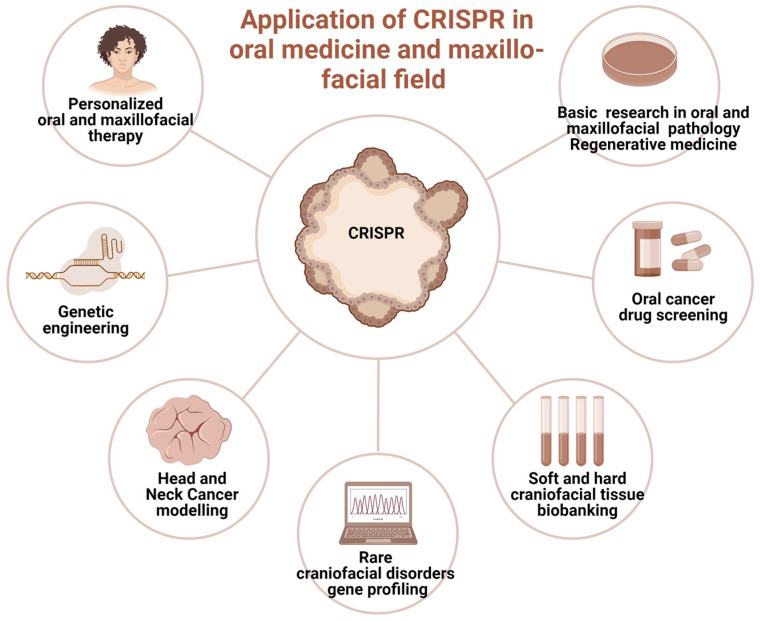
The prospective clinical applications of CRISPR genome-editing bioengineering in oral medicine and maxillofacial fields. Created in BioRender. Dziedzic, A. (2025) https://BioRender.com/6jtf5z8 (accessed on 1 January 2025).

Currently, sporadic reports of CRISPR-Cas clinical application in oral medicine provide scanty information primarily in the field of diagnosis, screening, and controlling oral diseases (Figure 3). They involve mitigation of viral diseases, head and neck cancer management, periodontitis management [37], and tissue regeneration [76]. The hypothetical opportunities of clinical utilization of GEBT CRISPR are deemed broad; however, preliminary studies do not allow for a precise prediction of the clinical indications for CRISPR/Cas9 genetics as a tool incorporated in clinical practice. Nevertheless, based on the unique features of precise genome modification, CRISPR may offer a promising alternative in certain hereditary or autoimmune conditions where conventional therapies have limited success, though this remains under preclinical investigation.

The future applications of gene editing biotechnology (GEBT) in oral and maxillofacial medicine are diverse and promising. Potential directions include personalized head and neck cancer screening and gene-based therapies for malignant and immune-related diseases, particularly hematological malignancies such as lymphoma, leukemia, and multiple myeloma, as well as conditions like AIDS that present with oral manifestations [77,78]. CRISPR may also enable individualized treatment of tumors arising from the maxillofacial region, including the development of therapeutic anticancer vaccines [79]. Tailored interventions for hereditary and developmental disorders with craniofacial involvement represent another key opportunity [80]. In regenerative surgery, xenotransplants facilitated by gene editing could enhance compatibility and outcomes in oral and maxillofacial reconstruction [81]. Furthermore, CRISPR/Cas9 holds potential in reprogramming somatic stem cells into induced pluripotent stem cells (iPSCs), which can be applied in tissue engineering strategies for craniofacial repair [81]. Finally, rapid CRISPR-based detection systems may improve diagnosis of oral diseases with microbial origins by enabling accurate and timely identification of viral and bacterial pathogens [38].

### 5.1. Primary Head and Neck Cancers and Oral Manifestations of Malignant Conditions

**Figure 4:**
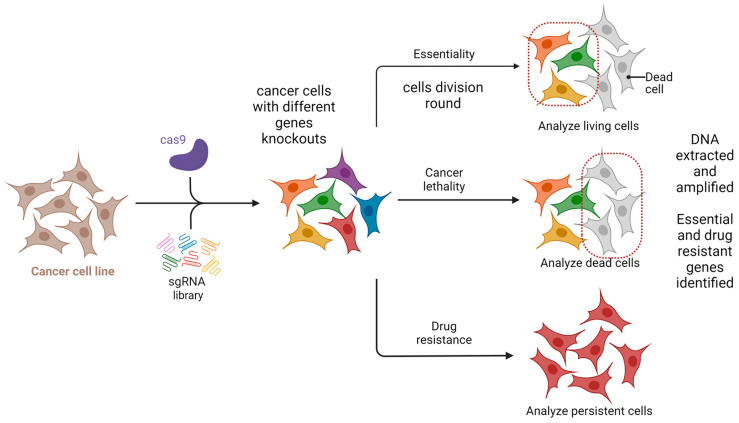
CRISPR-based screening for essentiality and drug resistance. Created in BioRender. Dziedzic, A. (2025) https://BioRender.com/9dh1tec (accessed on 1 February 2025).

One potential future application of CRISPR-based gene editing in oral oncology is the development of vaccines, particularly for HPV-related head and neck cancers. While promising in concept, such approaches remain in early experimental stages and are not yet supported by clinical data [38,39,40]. Selective use of such vaccines may also offer a precise therapeutic option for certain oral pathologies [38,39,40]. While CRISPR/GEBT is currently focused on treating hematologic malignancies like leukemia and multiple myeloma, its expanded application could reduce oral manifestations and mitigate the side effects of conventional chemotherapy [40,41]. A promising direction involves editing genes that drive carcinogenesis in the head and neck region. CRISPR/Cas9 holds transformative potential in this regard, particularly for targeting cancer stem cells (CSCs) harboring mutant p53 genes [42,43]. The technology is also being used to edit oncogenic microRNAs and to address drug resistance through disruption of the CD44 gene [44]. Additionally, CRISPR/Cas9 has been shown to reduce levels of urokinase plasminogen activator receptor, potentially halting tumor progression [44]. Preclinical studies support the efficacy of CRISPR/Cas9 in cancer models, and several clinical trials are underway to evaluate its therapeutic role [45]. The technology also shows promise in enhancing existing treatments like chemotherapy and targeted therapies. Drug resistance remains a major barrier in cancer therapy and involves genes related to drug efflux, DNA repair, apoptosis, and cellular signaling. Targeting these genes with CRISPR/Cas9 has shown potential to weaken resistance and improve drug response [42,43,44,45,46]. Head and neck cancers (HNC), known for high incidence and poor prognosis, may benefit from CRISPR-based therapies. HuR (ELAVL1), an RNA-binding protein that promotes tumor progression and resistance, has been effectively knocked out using CRISPR/Cas9. Combined with the chemotherapeutic agent epirubicin, HuR knockout in SAS cells triggered apoptosis, necroptosis, and autophagy, enhancing cancer cell death [38,39,40,41,42,43,44,45,46,47]. In vivo studies confirmed that using HuR-targeted CRISPR nanoparticles with epirubicin improved both efficacy and safety in treating HNC in mice [38,39,40,41,42,43,44,45,46,47]. Clinical trials are beginning to validate CRISPR applications in human cancer treatment. In trial NCT03081715, PD-1 genes were knocked out in peripheral blood lymphocytes from 16 esophageal cancer patients. Modified T cells were expanded ex vivo and reinfused. Each patient received between 1 and 10 × 10^9^ PD-1 knockout T cells. Treatment continued unless severe adverse effects emerged. Although completed in 2019, results are still pending [48]. An ongoing open-label, multicenter phase 1/2 trial (NCT05795595) is testing allogeneic CRISPR-edited CAR-T cells targeting CD70 in relapsed or refractory solid tumors, including esophageal cancer. This trial is expected to conclude by 2030 [48]. Further, CRISPR enables genetic engineering of T cells to enhance antitumor function. CISH (cytokine-induced SH2 protein), an intracellular checkpoint suppressing T cell activity, has been successfully inhibited via CRISPR without compromising cell viability. In trial NCT04426669, CISH-knockout T cells are being tested for safety and efficacy in patients with metastatic solid tumors, including esophageal cancer [49]. Figure 4 summarizes CRISPR-based screenings in cancer research, highlighting its role in neoadjuvant chemo- and immunotherapy for advanced head and neck squamous cell carcinoma [49].

### 5.2. Hereditary Oral and Maxilla- and Craniofacial Pathologies

**Figure 5:**
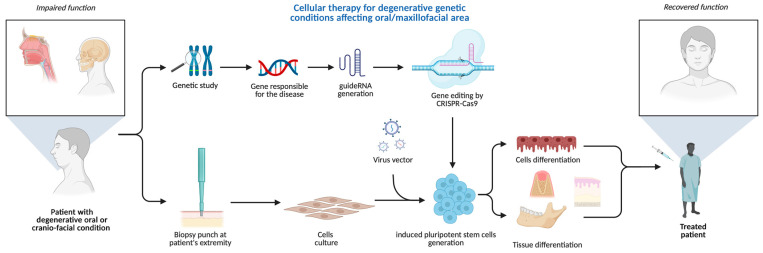
CRISPR-based cellular therapy for inherited conditions manifesting in oral/maxillofacial areas. Created in BioRender. Dziedzic, A. (2025) https://BioRender.com/h980ant (accessed on 5 August 2025).

The plethora of diseases and developmental defects affecting oral and maxillofacial areas, associated with genetic mutations, can exploit the groundbreaking CRISPR/GEBT. Considering the primary nature of some hereditary gene-related pathologies of hard and soft tissue, CRISPR/Cas seems unique and the only tool able to interfere with the human genome primarily in human cell lines and animal models [50]. A possible use of CRISPR technology in oral medicine is to correct or improve genetic defects that affect oral health, for example, to repair mutations that cause inherited disorders such as amelogenesis imperfecta, dentinogenesis imperfecta, or ectodermal dysplasia, which affect the development and structure of teeth and other oral tissues. Amelogenesis imperfecta is commonly caused by mutations in genes such as AMELX, ENAM, MMP20, and FAM83H, which are essential for enamel formation. Dentinogenesis imperfecta is primarily associated with mutations in the DSPP gene, which plays a key role in dentin structure and mineralization. Ectodermal dysplasia encompasses a group of conditions often linked to mutations in the EDA, EDAR, and EDARADD genes, which regulate the development of ectodermal structures, including teeth, hair, and sweat glands. CRISPR technology could also be used to enhance the expression of genes that promote bone regeneration, enamel remineralization, or wound healing [51] (Figure 5).

### 5.3. CRISPR-Based Xenotransplants in Maxillo-Facial Regenerative Surgery

**Figure 6:**
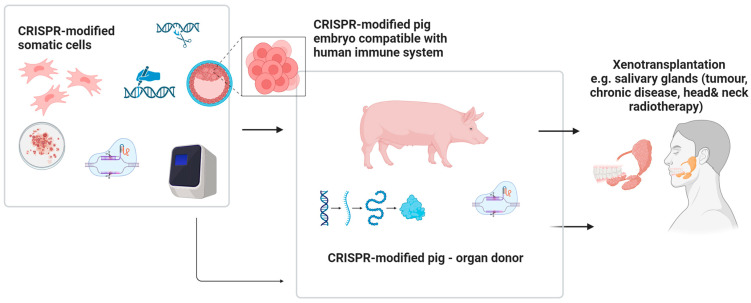
The future potential of the use of CRISPR facilitating xenogenic transplants of genetically modified tissues/organs within head and neck area. Created in BioRender. Dziedzic, A. (2025) https://BioRender.com/fnacht4 (accessed on 7 April 2025).

Recent clinical research indicates the promising application of GEBT/CRISPR technology in xenotransplantation, particularly in pig-to-human models [52]. Early studies in xenotransplantation suggest that CRISPR gene editing could improve compatibility of animal tissues for human use, but its application in oral and maxillofacial surgery remains highly experimental and speculative. CRISPR-mediated suppression of immune responses, even across significant phylogenetic distances, enhances the potential for successful integration of xenogeneic tissues. Hypothetical applications in the maxillofacial field could include reconstruction of facial bones following oncological resection or trauma, combined flap transfers, salivary gland transplantation, and the use of inert xenogeneic bone grafts for repairing osseous defects. However, as current biomedical research efforts largely focus on life-saving applications, the development of xenotransplantation for maxillofacial or orthognathic surgery may receive lower immediate priority [52] (Figure 6).

### 5.4. Application of the CRISPR/Cas9 System in Organoids and Tissue Engineering of Oral and Maxillofacial Diseases

Current treatment options for maxillofacial and oral diseases are mainly limited to surgical interventions, implants installment, artificial prostheses or autografts, which may not result in a successful restoration of function and/or esthetics. The application of organoids as a crucial component in regenerative medicine is gaining increasing scientific attention, with pluripotent stem cells (PSCs) and the chosen scaffolds, mainly those based on biopolymers such as collagen or embedded in hydrogels made of poly(lactic-co-glycolic acid) (PLGA) copolymers, leading the way. In the process of forming organoids, being 3D structures, exogenous factors play an important role, which trigger the differentiation of stem cells into a specific cell type [53,54,55]. This process can also be initiated using the CRISPR/Cas system to trigger the expression of a particular gene variant, which determines the induction of the differentiation process of cells into a particular cell type, as well as the generation of induced pluripotent stem cells (iPSCs) [55].

In a study by Ono et al. [56], generated iPSCs were used to test the regenerative potential of salivary gland cells. The study used embryonic submandibular gland (SG) cells, which were cocultured with iPSCs from mice. The results showed that these cells support each other’s differentiation, which was observed in the form of morphological changes that showed more bone marrow-like structures compared to monoculture. In addition, using molecular biology techniques, the team demonstrated that reduced expression levels of the key differentiation genes, *Sox2*, *c-Myc*, and *Nanong*, along with increased expression of *Klf4* and *Aqp5*, ultimately resulted in the regenerative potential of the salivary gland cells increasing. This suggests, therefore, that iPSCs that can be created by the CRISPR/Cas system offer broader possibilities in regenerative medicine for salivary gland-related diseases. A similar study demonstrating the positive effect of coculturing with iPSCs was conducted by Arakaki et al. [57] for enamel regeneration. They showed that coculturing mouse iPSCs with dental epithelial cells resulted in the differentiation of the cells into ameloblasts, which are crucial for enamel formation. Interestingly, they also showed a morphology like that of stromal cells, but did not express key markers for these cells, i.e., myeloblastic, emalin in the case of ameloblasts, and p63 and cytokeratin 14 as epithelial markers. This suggests that, despite the full similarity of the regenerated cells to their natural form, there are differences mainly at the molecular level, which should also be considered in future studies due to possible unwanted interactions with other cells. Currently a scanty information is available on the use of CRISPR/Cas9 in tissue regeneration in oral and maxillofacial diseases. Nevertheless, this system offers great opportunities in regenerative medicine, ranging from the reprogramming of stem cells to iPSCs and the correction of defective genes in selected genetically determined diseases [57].

### 5.5. Revolutionizing Pathogen Testing: Harnessing CRISPR for Rapid Detection of Specific Pathogens Responsible for Oral Diseases

While pathogen testing plays a critical role in safeguarding public health, it enables early detection and prevention of infectious diseases with oral manifestations. As traditional methods often suffer from limitations in terms of speed, accuracy, and accessibility, emerging CRISPR technology may become a promising tool for pathogen detection responsible for oral pathologies, particularly with viral origin [58]. Leveraging the exquisite precision of the CRISPR-associated Cas enzymes, the innovative CRISPR-based diagnostic platforms may offer rapid and specific identification of virulent pathogenic agents, important from an epidemiological perspective, such as highly transmissible and lethal viruses, including SARS-CoV-2 and potentially similar airborne microorganisms causing life-threatening infections, manifesting in the form of oral lesions [58]. There is a growing body of evidence that CRISPR has its potential applications, advancements, and prospects in transforming the field of infectious disease diagnosis.

**Figure 7:**
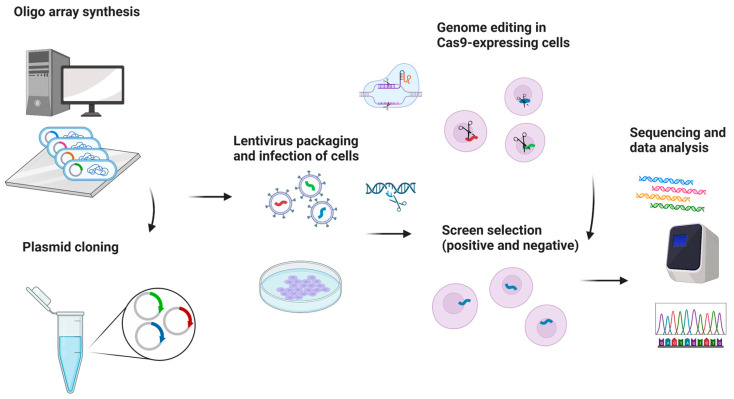
Harnessing CRISPR for rapid pathogens detection. Created in BioRender. Dziedzic, A. (2025) https://BioRender.com/tc160np (accessed on 1 June 2025).

Accurate and timely detection of pathogens is crucial for effective disease control, outbreak management, and the development of targeted therapeutic interventions. Traditional pathogen testing methods, such as polymerase chain reaction (PCR) and culture-based techniques, have provided significant contributions to public health. However, these methods often suffer from drawbacks, including time-consuming workflows, limited sensitivity, and dependency on specialized laboratory infrastructure. In recent years, the revolutionary CRISPR technology has emerged as a versatile and powerful tool that has revolutionized various areas of biology and biotechnology. Harnessing the inherent specificity and programmability of CRISPR-associated nucleases, such as Cas9, researchers have devised innovative strategies for pathogen detection that offer improved speed, accuracy, and accessibility [59,60]. CRISPR-based pathogen detection platforms generally operate through a two-step mechanism. First, a guide RNA (gRNA) specifically binds to a complementary target sequence in the pathogen’s nucleic acids. Second, the Cas enzyme, directed by the gRNA, induces site-specific cleavage or modification, resulting in a measurable signal [61]. This signal may be amplified and detected through various analytical techniques, including fluorescence, lateral flow assays, and next-generation sequencing. The inherent programmability of CRISPR systems enables rapid customization of gRNAs to target specific sequences, supporting the development of highly specific and sensitive diagnostic assays. Recent advances have demonstrated the utility of CRISPR-based diagnostics for detecting a broad range of pathogens, including bacteria, viruses, and parasites [58,59,60,61,62,63]. Additionally, portable and field-deployable CRISPR diagnostic devices have shown potential in resource-limited environments and outbreak scenarios, offering rapid, on-site detection and surveillance. The integration of CRISPR with complementary amplification techniques, such as loop-mediated isothermal amplification (LAMP), has further improved assay sensitivity and operational efficiency in point-of-care contexts [62]. Despite these advances, further research is needed to optimize assay sensitivity, enable multiplexing, and streamline sample preparation. The successful clinical implementation of CRISPR-based diagnostics will also require resolution of issues related to standardization, regulatory approval, and cost-effectiveness [63,64]. Ongoing collaboration among researchers, clinicians, and policymakers will be essential to support the translation of these technologies into routine clinical use for accurate and timely identification of infectious agents (Figure 7).

### 5.6. Common Oral Diseases: Periodontitis and Dental Caries

CRISPR-based genome editing technologies (GEBT) are under investigation for their potential role in improving the diagnosis and treatment of chronic oral inflammatory diseases, including periodontitis. Emerging evidence suggests that CRISPR-Cas9 knockout systems can be used to investigate molecular pathways involved in periodontal disease pathogenesis [65]. CRISPR-Cas systems enable targeted genetic modification, including the removal of specific DNA sequences, gene disruption, and regulation of gene expression. These capabilities are being explored for applications in the management of oral and periodontal diseases, particularly in developing precision-based therapeutic strategies [82]. Barbour et al. [66] proposed that selected CRISPR systems (e.g., Cas13 and Cas3) could be applied in personalized periodontal care to modulate gene expression associated with disease onset and progression. Moreover, CRISPR-based approaches may offer control over pathogenic biofilms implicated in periodontal infections. For example, deletion of the cas3 gene has been shown to induce expression of genes involved in reactive oxygen species production and iron acquisition in bacteria, contributing to a pro-inflammatory environment favorable to periopathogen survival. A meta-transcriptomic study by Solbiati et al. [67] reported a seventeen-fold increase in cas3 gene expression in sites transitioning from health to disease, underscoring its potential role in microbial dysbiosis and periodontal inflammation.

## 6. Exploring the Frontiers: Unveiling the Constraints and Challenges of GEBT

Despite the revolutionary potential of CRISPR-based gene editing in oral and maxillofacial medicine, several key limitations and ethical concerns continue to restrict its clinical translation. These include procedural complexity, technical limitations, safety concerns, economic factors, and moral objections, particularly in pediatric and dental applications. A focused examination of these barriers is critical to understanding the feasibility and future direction of CRISPR technology in dental and craniofacial care.

### 6.1. Procedural and Technical Challenges

CRISPR applications demand specialized bioengineering infrastructure, highly trained personnel, and access to sophisticated molecular tools. Many dental institutions and maxillofacial centers lack the interdisciplinary resources required for implementing these procedures. Unlike routine dental therapies, CRISPR involves high-stakes manipulation of the genome, requiring stringent controls for guide RNA design, delivery mechanisms, and validation protocols [65,72,82]. A central technical issue is the risk of off-target mutations, where unintended regions of DNA are edited, potentially resulting in harmful effects such as carcinogenesis or immunogenicity [15,17,70,82]. While Cas9 variants and improved guide RNA algorithms help reduce this risk, the technology is not yet foolproof. These concerns are especially pressing when considering genome editing in oral tissues with regenerative properties, such as dental pulp stem cells, where unintended edits may have prolonged or irreversible effects [57,77,81].

### 6.2. In Vivo vs. Ex Vivo Delivery: Dental Relevance

The distinction between in vivo and ex vivo gene editing is particularly important when considering dental and oral tissues. Ex vivo editing, where cells are modified outside the body and reintroduced, offers greater control and monitoring, making it more suitable for applications like engineering autologous dental pulp stem cells or bone marrow stromal cells for jawbone regeneration [40,55]. These applications are promising in treating craniofacial anomalies or periodontitis-related bone loss [76,80,83]. In contrast, in vivo editing, while less invasive, poses higher risks due to limited control over delivery precision and biological responses. Systemic delivery of CRISPR components into the oral cavity, such as salivary glands, mucosa, or alveolar bone, faces challenges like enzyme degradation, uneven tissue distribution, and unintended immune activation [70,71]. Therefore, current oral health applications are likely to favor ex vivo protocols in the near term, especially in the pediatric population or in cases involving congenital craniofacial syndromes [50,53].

### 6.3. Economic and Infrastructure Barriers

Gene editing technologies are expensive. Costs stem not only from the gene editing kits themselves but also from the infrastructure needed to support the technology: sequencing platforms, biocontainment labs, and clinical-grade manufacturing. This economic burden severely limits access in low- and middle-income countries and even within resource-limited public healthcare systems in developed nations [72,73]. Moreover, there are overlapping concerns regarding intellectual property and commercial licensing that hinder open-access development [74]. While CRISPR offers long-term cost benefits through disease prevention, its short-term expense can prevent equitable implementation, especially in oral health, which already receives lower prioritization in public health funding relative to systemic or life-threatening diseases [75].

### 6.4. Ethical Concerns in Oral and Pediatric Gene Editing

The ethical challenges of genome editing extend beyond general medicine into the specific domain of oral healthcare. In particular, pediatric applications raise questions of consent, long-term safety, and the justification for early-life genetic modification [68,69]. Pediatric craniofacial conditions, such as cleft lip/palate or ectodermal dysplasias, could theoretically benefit from early CRISPR-based interventions, but these applications are ethically fraught due to the irreversible nature of the intervention and the inability of minors to provide informed consent. Similarly, the use of dental stem cells for gene editing, especially those derived from deciduous teeth or wisdom teeth, raises issues of biobanking, autonomy, and future use without patient awareness [53,55]. Additionally, concerns about “enhancement vs. therapy” are particularly salient in dental medicine, where the line between therapeutic necessity (e.g., correcting cleft palate genes) and esthetic enhancement (e.g., tooth color, enamel hardness) may blur [78,81]. From a bioethical standpoint, these concerns require robust policy frameworks, transparent informed consent processes, and possibly age-specific guidelines that recognize the special vulnerability of pediatric dental patients. Regulatory oversight must also ensure that oral CRISPR applications meet the same rigorous safety and efficacy standards as in systemic medicine [71].

The pitfalls and limitations of GEBT are considerable and multifaceted. The technique is highly sensitive to laboratory and procedural protocols, as CRISPR requires advanced skills in molecular bioengineering that exceed routine laboratory medicine, demanding precise execution by specialized experts [81]. Financial constraints also pose major barriers since the technology involves costly equipment, high processing expenses, and licensing fees, which hinder widespread adoption [66]. Effective application further necessitates close interdisciplinary cooperation among clinicians, scientists, geneticists, and bioengineering specialists [67]. There is also a risk of procedural errors if results are not carefully validated and controlled [68]. Establishing reliable diagnostic or therapeutic targets can be difficult, especially for oral medicine and maxillofacial conditions with unknown etiologies [69]. Additionally, many therapeutic protocols remain experimental and at early stages of clinical trials, carrying the risk of systemic side effects or off-target mutations where complications may outweigh benefits [69,72]. The absence of long-term outcome data contributes to unpredictable effects and possible trial failures [73,74]. Moreover, the highly individualized nature of therapeutic aims may not be broadly applicable across populations, raising questions about cost-effectiveness when public health resources are considered [70,75,84].

## 7. Future Directions and Research Priorities

While CRISPR/Cas9 technology holds clear potential for oral and maxillofacial medicine, its future success depends on careful prioritization of research efforts, standardized outcome assessment, and realistic integration into existing clinical frameworks. Rather than assuming linear progress, it is essential to establish a roadmap grounded in translational relevance, ethical feasibility, and cost-effectiveness.

### 7.1. Priority Areas for Clinical Research

Based on current gaps and feasibility, the following four areas represent the most promising and clinically relevant domains for CRISPR research in oral health: gene therapy for congenital craniofacial disorders [3,75], focusing on monogenic, structurally manifesting conditions such as amelogenesis imperfecta, dentinogenesis imperfecta, and ectodermal dysplasia, which offer clear genotype-phenotype relationships and can be modeled using organoids and dental pulp stem cells; CRISPR in oral cancer immunotherapy [41] (Table 3) building on ongoing oncology trials by testing CRISPR-modified T cells in HPV-positive oral squamous cell carcinoma or nasopharyngeal cancer, with potential for combination therapies involving CAR-T or immune checkpoint inhibitors; regenerative dentistry using ex vivo gene-edited cells, aimed at engineering patient-derived stem cells to regenerate bone, enamel, or salivary tissue, especially for post-oncologic or traumatic reconstruction, with an emphasis on controlled ex vivo models to reduce systemic risk; and CRISPR-based diagnostics for oral pathogens, which seek to advance rapid, chair-side detection platforms like SHERLOCK and DETECTR for identifying infections caused by HPV, EBV, and periodontal pathogens, offering a more immediate path to clinical application compared to therapeutic editing.

Based on current knowledge gaps, priority areas for future research include the development of standardized delivery systems tailored to oral tissues such as salivary glands, pulp stem cells, and craniofacial bone; longitudinal studies designed to assess the durability and safety of CRISPR interventions in relevant preclinical models; expansion of clinical trials targeting common oral diseases such as periodontitis and caries, which remain underexplored despite their global burden; and comparative evaluations of CRISPR-based diagnostics against conventional platforms with respect to accuracy, turnaround time, and cost-effectiveness.

### 7.2. Metrics for Evaluating CRISPR in Oral Medicine

To assess the meaningful progress of CRISPR applications in the dental field, objective and multidisciplinary metrics should be applied. Suggested criteria include the clinical safety profile, which considers the frequency and severity of adverse effects such as off-target events and immune reactions in oral tissue-specific models or trials, with metrics distinguishing between in vivo and ex vivo interventions. Targeting precision and efficiency should be measured by the percent success in achieving intended gene edits without off-target mutations, validated through deep sequencing in tissue-specific CRISPR workflows. Functional outcomes such as restoration of tooth structure, bone integrity, glandular secretion, or tumor regression should be evaluated using standardized dental indices like the DMFT score, bone fill percentage, and histopathology. Translational scalability refers to the ability to reproduce results across patient cohorts, integrate them into existing clinical workflows, and achieve cost-effective scalability in real-world settings. Finally, ethical and social acceptability must be considered by assessing patient willingness to accept genome editing, particularly in pediatric populations or for elective esthetic purposes, with qualitative studies and ethical review audits incorporated early in protocol development. Recent studies have highlighted the potential of mesenchymal stem cells (MSCs) derived from human periapical cysts as a promising resource for regenerative applications in oral and maxillofacial medicine [84]. These periapical cyst–derived MSCs exhibit multipotency and immunomodulatory capabilities comparable to other dental MSC sources but originate from readily accessible pathological tissues. Notably, research has identified differential gene expression profiles between MSCs from periapical cysts and those from granulomatous tissue, particularly in genes related to mineralization and dentinogenesis [71,85,86]. In particular, the expression levels of Dentin Matrix Acid Phosphoprotein 1 (DMP-1) and Dentin Sialophosphoprotein (DSPP), both essential for dentin formation, are altered, suggesting distinct differentiation capacities. The integration of CRISPR-based genome editing with these unique stem cell populations may provide a platform for targeted gene correction or enhancement of odontogenic potential. Future research should explore how CRISPR-mediated modulation of lineage-specific genes in these MSCs could improve outcomes in bone and dentin regeneration therapies, potentially expanding the clinical utility of otherwise discarded periapical tissues.

### 7.3. Limitations of This Study

The limitations of this review can be categorized into three domains: methodological, evidentiary, and clinical translation. Methodologically, the absence of a formal risk of bias assessment and reliance on narrative synthesis limit internal validity and increase susceptibility to selection or interpretive bias. From an evidentiary standpoint, most included studies were preclinical, small-scale, or dependent on surrogate endpoints, with substantial heterogeneity in targets, vectors, and outcomes. Publication bias remains a concern in gene editing research, as early experimental studies are more likely to report positive or proof-of-concept outcomes, whereas negative or null findings may be underrepresented in the literature. In terms of clinical translation, no completed clinical trial has yet demonstrated therapeutic efficacy in common oral diseases, and the lack of standardized delivery systems, cost-effectiveness analyses, and long-term safety monitoring continues to constrain applicability to routine practice.

This scoping review was designed to chart breadth rather than quantify treatment effects, which precludes causal inference and comparative effectiveness estimates and limits external validity to routine oral and maxillofacial practice. The evidence base is heavily preclinical, with predominance of in vitro systems, organoids, and animal models, and only a small number of early phase clinical studies that are not powered for efficacy, which constrains translational generalizability. Marked heterogeneity in gene targets, delivery vectors, editing modalities, model systems, and outcome measures prevented meta-analysis and necessitated narrative synthesis that is susceptible to selection and interpretive bias.

Many included reports emphasize feasibility, analytical validity, or surrogate endpoints rather than patient-centered outcomes such as pain reduction, functional restoration, salivary flow, quality of life, or long-term safety, and few provide standardized adverse event adjudication, off-target detection with high sensitivity, dose rationale, or pharmacovigilance beyond short follow-up. Small sample sizes, interim analyses, incomplete outcome reporting, and censoring further limit precision and reproducibility. The current clinical trial landscape is skewed toward systemic oncology and hematology, which may overrepresent immuno-oncology paradigms and underrepresent common oral diseases such as periodontitis and caries, thereby limiting immediate applicability to maxillofacial care pathways and health economic evaluation. Finally, rapid iteration in CRISPR engineering, delivery systems, and regulatory frameworks means that conclusions may become outdated as new data emerge beyond November 2024.

We did not conduct a formal risk of bias appraisal of individual studies, consistent with the aims of a scoping review; therefore, study quality was not graded, and our findings should be interpreted as evidence mapping rather than effect estimation. The absence of a standardized risk of bias assessment may introduce interpretive uncertainty, so results are presented to characterize the landscape and not to support comparative claims about effectiveness.

### 7.4. Limitations of Evidence

Despite increasing interest in CRISPR applications for oral and maxillofacial conditions, the current body of evidence remains limited and uneven. Most available studies are preclinical, relying on in vitro models, animal research, or conceptual frameworks, with only a small number of early-phase clinical trials relevant to oral pathology. High-quality, peer-reviewed clinical studies specific to dental or craniofacial applications are scarce. Furthermore, the heterogeneity in study designs, gene targets, delivery methods, and outcome measures makes it difficult to compare results or draw generalizable conclusions. Evidence supporting CRISPR’s use in common oral diseases such as periodontitis or dental caries is largely theoretical, with few translational studies available. This evidentiary gap underscores the need for rigorous clinical research and standardized protocols tailored to the unique challenges of oral and craniofacial medicine. This review identified 22 registered clinical trials investigating CRISPR-based therapies (Table S1). While these trials confirm that CRISPR has entered the clinical arena, their scope is almost exclusively systemic or oncologic, with limited direct relevance to oral or maxillofacial conditions. Most are early-phase studies (Phase 1 or 1/2), designed to evaluate feasibility and safety rather than therapeutic efficacy. Importantly, no completed trial to date has reported validated outcomes in common oral diseases such as periodontitis, caries, or craniofacial developmental disorders. Thus, despite the growing number of registered trials, the clinical evidence base for CRISPR in oral medicine remains preliminary and indirect, underscoring the speculative nature of many projected applications.

### 7.5. Long-Term Considerations

Rather than expecting immediate clinical translation, the future of CRISPR in oral health may follow a tiered model: initial use in laboratory settings and ex vivo proof-of-concept studies, followed by compassionate use cases in refractory genetic or neoplastic diseases, and eventual incorporation into standard care for select indications. Governmental and academic funding should prioritize CRISPR-based interventions that address unmet therapeutic needs, demonstrate cost-effectiveness, and align with public health objectives. Meanwhile, global efforts to establish standardized CRISPR regulatory frameworks for dental and maxillofacial medicine must proceed in parallel.

Although the evidence base is expanding, study quality is uneven and often limited by small sample sizes, short follow-up, and incomplete reporting of adverse events and off-target assays. Marked heterogeneity in gene targets, delivery vectors, editing modalities, models, and outcome definitions reduces comparability and precludes pooled estimates. Many reports prioritize feasibility or surrogate endpoints over patient-centered outcomes such as pain, function, salivary flow, or quality of life, which weakens clinical relevance. Off-target risk assessment remains inconsistent across studies, and detection methods vary in sensitivity, which complicates safety benchmarking. Translational barriers are substantial, including delivery constraints in oral tissues, immunogenicity, manufacturing complexity, regulatory uncertainty, and high costs that may limit equitable access. Together, these factors suggest that CRISPR applications in oral and maxillofacial care remain at early technology readiness levels, with clinical translation contingent on standardized protocols, robust safety analytics, and reproducible functional outcomes.

The trajectory of ongoing trials suggests that preliminary readouts from early-phase oncology studies (e.g., PD-1 knockout T cells, CRISPR-edited CAR-T therapies) may emerge within the next 3–5 years, potentially informing safety benchmarks applicable to oral oncology. Advances in delivery technologies, including lipid nanoparticles, viral vectors with tissue tropism, and CRISPR base/prime editors with reduced off-target risk, are expected to accelerate translation over the coming decade. For oral health applications, ex vivo editing of dental pulp or salivary stem cells may become technically feasible within a similar timeframe, whereas in vivo delivery for hereditary craniofacial syndromes is likely a long-term (>10 years) goal. Successful integration of CRISPR into oral and maxillofacial practice will require changes that extend beyond technical research progress. Regulatory frameworks must evolve to address dental- and craniofacial-specific applications, balancing innovation with rigorous oversight of off-target risks and ethical considerations. Commercial pathways, including cost reduction of editing platforms, streamlined licensing, and public–private partnerships, will be essential to ensure equitable access. Educational initiatives for dental clinicians and surgeons are also needed to build genomic literacy and prepare practitioners for the potential incorporation of gene editing strategies into patient care. Without coordinated advances in these regulatory, commercial, and educational domains, clinical translation will remain fragmented even if scientific feasibility improves.

### 7.6. Key Takeaways: CRISPR in Oral and Maxillofacial Medicine

- Current Use Is Preliminary: Most CRISPR-related clinical trials target systemic conditions. Common or rare oral and maxillofacial applications remain largely preclinical or conceptual.
- Most Feasible Short-Term Applications: CRISPR-based diagnostics for oral pathogens (e.g., HPV, EBV). Ex vivo editing of dental stem cells for regenerative therapy. Precision oncology using CRISPR-engineered T cells in head and neck cancers.
- Long-Term Potential Areas: Gene therapy for hereditary enamel/dentin defects (e.g., amelogenesis/dentinogenesis imperfecta). Personalized immunotherapy for oral cancers. Integration with tissue engineering and organoids for craniofacial regeneration.
- Primary Barriers to Implementation: Off-target mutation risk and immune responses. High cost and limited access to gene editing platforms. Ethical concerns in pediatric and elective contexts. Lack of standard protocols for oral tissue targeting.

## 8. Conclusions

Given the quality and heterogeneity of the current evidence, immediate routine clinical use in oral and maxillofacial practice is premature. Near-term impact is most plausible for CRISPR-based diagnostics and ex vivo edited cell products evaluated within controlled protocols; in vivo therapeutic editing should remain confined to ethically approved clinical trials. Progress will depend on harmonized safety assays for off-target events, standardized functional endpoints relevant to dental care, and cost-effective manufacturing pathways that support equitable access. Critical gaps include the lack of standardized delivery systems for oral tissues, limited long-term safety data, and insufficient translational models that reflect real-world disease complexity. Ethical concerns, high costs, and regulatory uncertainties further constrain its immediate applicability. While CRISPR shows potential in areas such as diagnostics, regenerative strategies, and oncology, these applications should be regarded as exploratory rather than established. Future progress depends on generating rigorous clinical evidence, addressing methodological weaknesses, and realistically assessing feasibility before integration into oral healthcare.

## Figures and Tables

**Table 1:** Overview of Key CRISPR Mechanisms in Clinical Gene Editing.

Component	Function	Clinical Relevance
sgRNA	Directs Cas9 to DNA sequence	Determines targeting precision
Cas9	Endonuclease that cuts DNA	Enables therapeutic gene disruption/correction
PAM (Protospacer Adjacent Motif)	DNA motif required for Cas9 activity	Limits off-target binding
NHEJ	Error-prone repair; introduces mutations	Used to silence harmful genes
HDR	Precise repair using a template	Used for correcting specific mutations

**Table 2:** Clinical applications of CRISPR in oral/maxillofacial medicine.

Condition/Domain	CRISPR Application	Status	References
Oral Squamous Cell Carcinoma	Oncogene knockout (e.g., p53, PD-1), CAR-T therapy	Preclinical and early trials	[5,6,42,43,45]
HPV-related Head and Neck Cancers	Immunotherapy, PD-1 knockout, therapeutic vaccines	Phase 1/2 trials	[38,39,48,49]
Craniofacial Syndromes (e.g., AI, DI)	Gene correction via CRISPR-Cas9	Preclinical (iPSC/animal models)	[3,50,51]
Periodontitis	Gene pathway targeting, biofilm modulation	Exploratory preclinical	[65,66,67]
Salivary Gland Regeneration	Organoid modeling, iPSC regeneration	Preclinical studies	[40,53,56]
Oral Viral Infections (e.g., HPV, HIV)	Viral gene targeting, CRISPR-mediated immunity	Ongoing preclinical and trial development	[35,36,58]
Pathogen Detection (e.g., SARS-CoV-2, EBV)	CRISPR diagnostics (e.g., SHERLOCK, DETECTR)	Proof-of-concept and rapid testing prototypes	[58,61,62,63,64]

**Table 3:** Preclinical models and outcomes: ethical, technical, and economic limitations.

Model/System	CRISPR Target	Outcome	References
Murine models of oral cancer	p53, CD44, HuR, PD-1	Tumor suppression, chemo-sensitization	[42,43,44,47]
iPSC-derived ameloblast cultures	AMELX, ENAM, FAM83H	Partial enamel regeneration, ameloblast-like cell differentiation	[50,57]
Coculture of iPSCs and salivary gland cells	Sox2, Nanog, Aqp5	Improved epithelial regeneration in salivary organoids	[56]
Porphyromonas gingivalis gene knockout	cas3	Reduced bacterial virulence and inflammation control	[66,67]
Craniofacial bone regeneration (mouse model)	BMP, RUNX2-related pathways	Enhanced bone formation and osteogenic differentiation	[53,54,55]

**Table 4:** Ethical, technical, and economic limitations.

Category	Challenges	Implications	References
Ethical	Pediatric consent, germline risk, enhancement vs. therapy	Requires tailored ethics protocols and public debate	[8,68,69]
Technical	Off-target effects, delivery inefficiency, repair unpredictability	Need for improved Cas9 variants, better vectors	[13,15,16,70,71]
Economic	Cost of CRISPR tools, equipment, and licensing	Limited access in low-resource settings; scalability challenges	[72,73,74,75]

**Table 5:** Future directions grouped by feasibility.

Feasibility Level	Focus Area	Rationale	References
High (near-term)	CRISPR-based diagnostics for oral pathogens	Low risk, portable, high specificity	[58,61,62,63,64]
Moderate	Ex vivo stem cell editing for regenerative therapy	Controlled editing, compatible with dental workflows	[53,55,56,57]
Low (long-term)	In vivo gene therapy for hereditary craniofacial disorders	Ethical and technical hurdles, complexity of delivery	[3,50,51,68]

## Data Availability

All data generated is presented in this document.

## Supplementary Materials

The following supporting information can be downloaded at: https://www.mdpi.com/article/10.3390/biomedicines13112745/s1, Table S1: Search Strategy for CRISPR-related Research in Oral and Craniofacial Domains Across Databases.

## Abbreviations

The following abbreviations are used in this manuscript:

3D	Three-dimensional
AIDS	Acquired Immunodeficiency Syndrome
AI	Amelogenesis Imperfecta
AMELX	Amelogenin X-linked gene
AQP5	Aquaporin 5
BMP	Bone Morphogenetic Protein
CAR-T	Chimeric Antigen Receptor T cells
Cas	CRISPR-associated protein
Cas9	CRISPR-associated Endonuclease 9
Cas12	CRISPR-associated Endonuclease 12
Cas13	CRISPR-associated Endonuclease 13
CD19	Cluster of Differentiation 19
CD33	Cluster of Differentiation 33
CD44	Cluster of Differentiation 44
CD70	Cluster of Differentiation 70
CISH	Cytokine Inducible SH2-Containing Protein
CRISPR	Clustered Regularly Interspaced Short Palindromic Repeats
CRISPR/Cas	CRISPR with CRISPR-associated Protein System
DMP1 or DMP-1	Dentin Matrix Protein 1
DMFT	Decayed, Missing, and Filled Teeth Index
DETECTR	DNA Endonuclease-Targeted CRISPR Trans Reporter
DI	Dentinogenesis Imperfecta
DSPP	Dentin Sialophosphoprotein
EBV	Epstein–Barr Virus
EDA	Ectodysplasin A gene
EDAR	Ectodysplasin A Receptor gene
EDARADD	EDAR-Associated Death Domain gene
ELAVL1 or HuR	ELAV-like RNA-Binding Protein 1
ENAM	Enamelin Gene
FAM83H	Family With Sequence Similarity 83 Member H gene
gRNA	Guide RNA
GEBT	Gene Editing Biotechnology
HDR	Homology Directed Repair
HLA	Human Leukocyte Antigen
HNC	Head and Neck Cancer
HNSCC	Head and Neck Squamous Cell Carcinoma
HNH	Histidine–Asparagine–Histidine Nuclease Domain of Cas9
HPV	Human Papillomavirus
HSC	Hematopoietic Stem Cell
iPSC	Induced Pluripotent Stem Cell
INDELs	Insertions and Deletions
LAMP	Loop-mediated Isothermal Amplification
MeSH	Medical Subject Headings
MMP20	Matrix Metallopeptidase 20
MSC	Mesenchymal Stem Cell
MUC1	Mucin 1
NCT	National Clinical Trial identifier
NHEJ	Non-homologous End Joining
OSCC	Oral Squamous Cell Carcinoma
PAM	Protospacer Adjacent Motif
PBMC	Peripheral Blood Mononuclear Cell
PCR	Polymerase Chain Reaction
PD-1	Programmed Cell Death Protein 1
PDCD1	Programmed Cell Death 1 gene
PDL-1 or PD-L1	Programmed Death Ligand 1
PLGA	Poly(lactic-co-glycolic acid)
PRISMA-ScR	Preferred Reporting Items for Systematic Reviews and Meta-Analyses Extension for Scoping Reviews
PSC	Pluripotent Stem Cell
RNP	Ribonucleoprotein
RUNX2	Runt-related Transcription Factor 2
RuvC	RuvC-like nuclease domain of Cas9
SARS-CoV-2	Severe Acute Respiratory Syndrome Coronavirus 2
sgRNA	Single-guide RNA
SHERLOCK	Specific High-sensitivity Enzymatic Reporter unLOCKing
SG	Salivary Gland
Sox2	SRY-box Transcription Factor 2
TALEN	Transcription Activator-like Effector Nuclease
TB	Tuberculosis
TCR	T Cell Receptor
TILs	Tumor-Infiltrating Lymphocytes
uPAR	Urokinase-type Plasminogen Activator Receptor
WT1	Wilms Tumor 1 gene
ZFN	Zinc Finger Nuclease
