# Supplementary material for: CRISPR Genome Editing in Personalized Therapy for Oral and Maxillofacial Diseases: A Scoping Review
Source: Biomedicines. 2025 Nov 10;13(11):2745. doi: 10.3390/biomedicines13112745 (PMC12650159; doi:10.3390/biomedicines13112745)
Supplement: Supplementary file 1 [file biomedicines-13-02745-s001.zip › biomedicines-3911244-supplementary.pdf]

Table S1. Search Strategy for CRISPR-related Research in Oral and Craniofacial Domains Across Databases.

Database	Search string	Notes/Adaptations
PubMed/MEDLINE	("CRISPR" OR "CRISPR Cas9" OR "Cas12" OR "Cas13" OR "base editing" OR "prime editing") AND ("oral" OR "dent*" OR "periodont*" OR "maxillofacial" OR "craniofacial" OR "salivary")	Search limited to English language, 2012–2024. MeSH terms were incorporated where available.
Scopus	TITLE-ABS-KEY ("CRISPR" OR "CRISPR Cas9" OR "Cas12" OR "Cas13" OR "base editing" OR "prime editing") AND TITLE-ABS-KEY ("oral" OR "dent*" OR "periodont*" OR "maxillofacial" OR "craniofacial" OR "salivary")	Syntax adapted for Scopus advanced search; applied filters for subject areas Medicine, Dentistry, Biochemistry, Genetics.
Web of Science	TS=("CRISPR" OR "CRISPR Cas9" OR "Cas12" OR "Cas13" OR "base editing" OR "prime editing") AND TS=("oral" OR "dent*" OR "periodont*" OR "maxillofacial" OR "craniofacial" OR "salivary")	Limited to 2012–2024, English.
ClinicalTrials.gov	Condition/Disease: ("oral" OR "dent*" OR "periodont*" OR "maxillofacial" OR "craniofacial" OR "salivary") AND Other terms: ("CRISPR" OR "CRISPR Cas9" OR "Cas12" OR "Cas13" OR "gene editing" OR "base editing" OR "prime editing")	Search restricted to interventional studies; includes both recruiting and completed trials.
